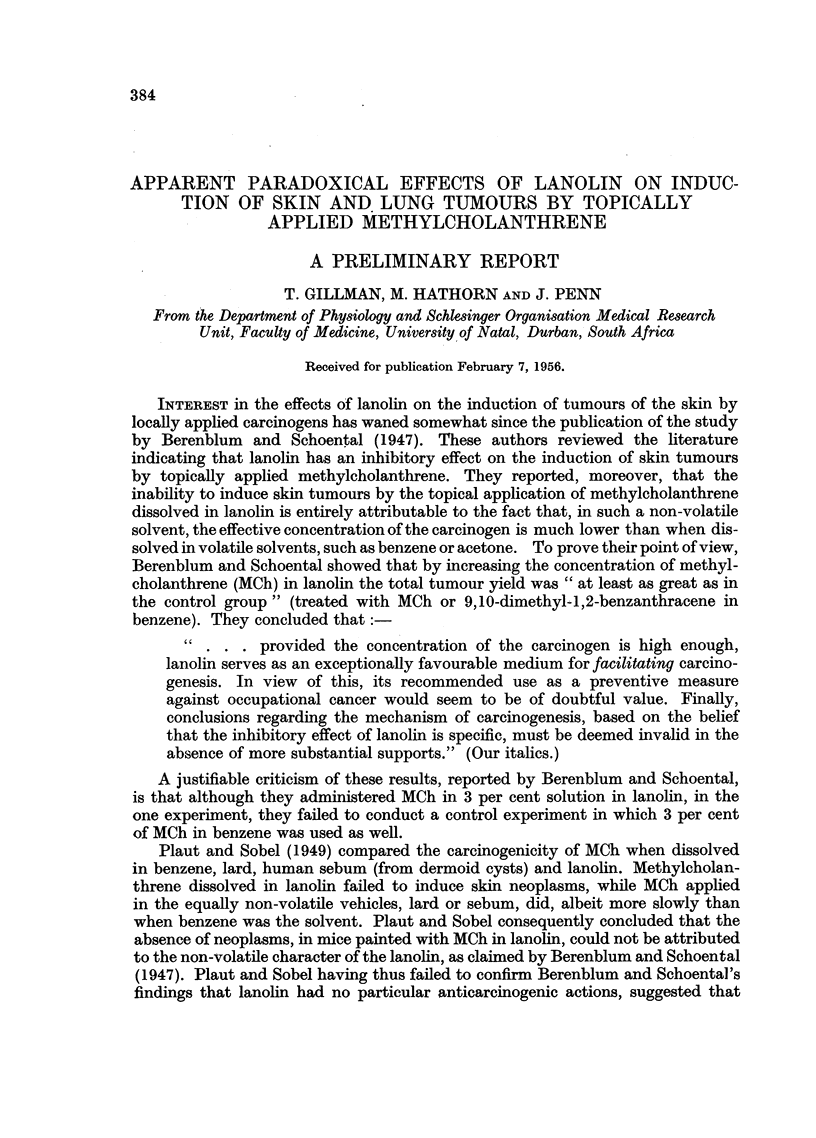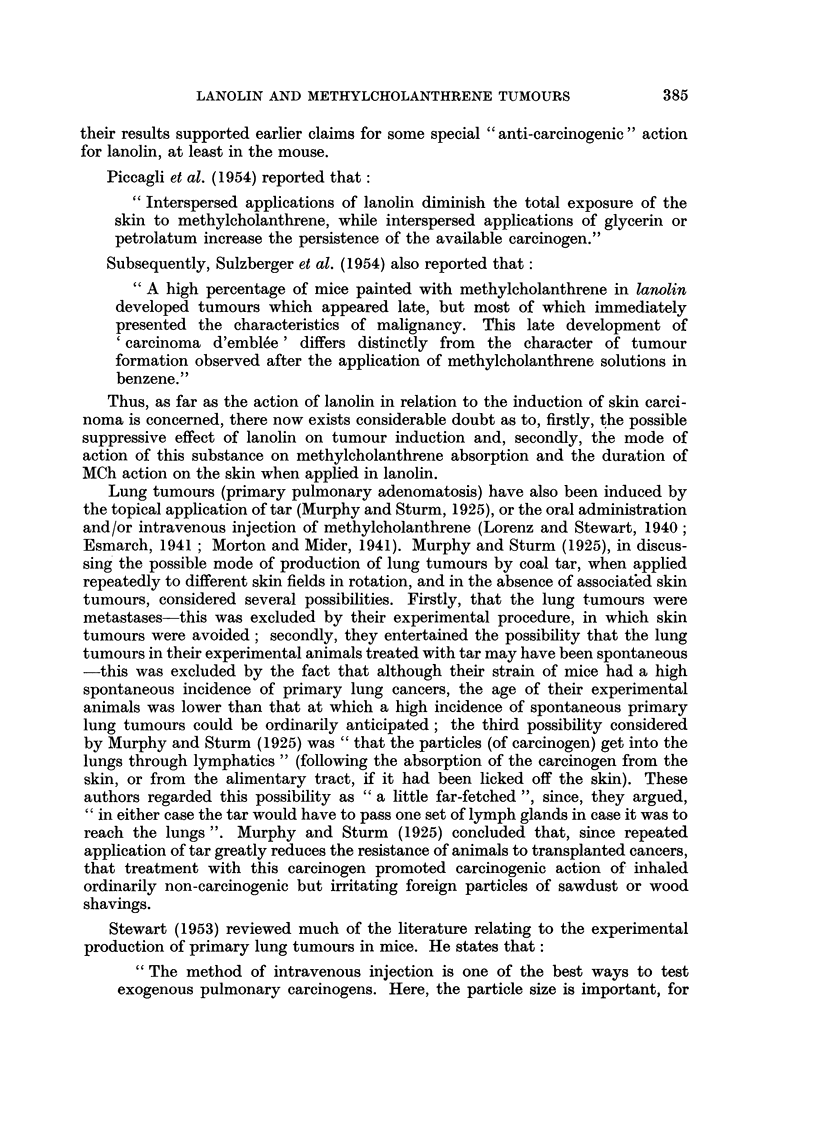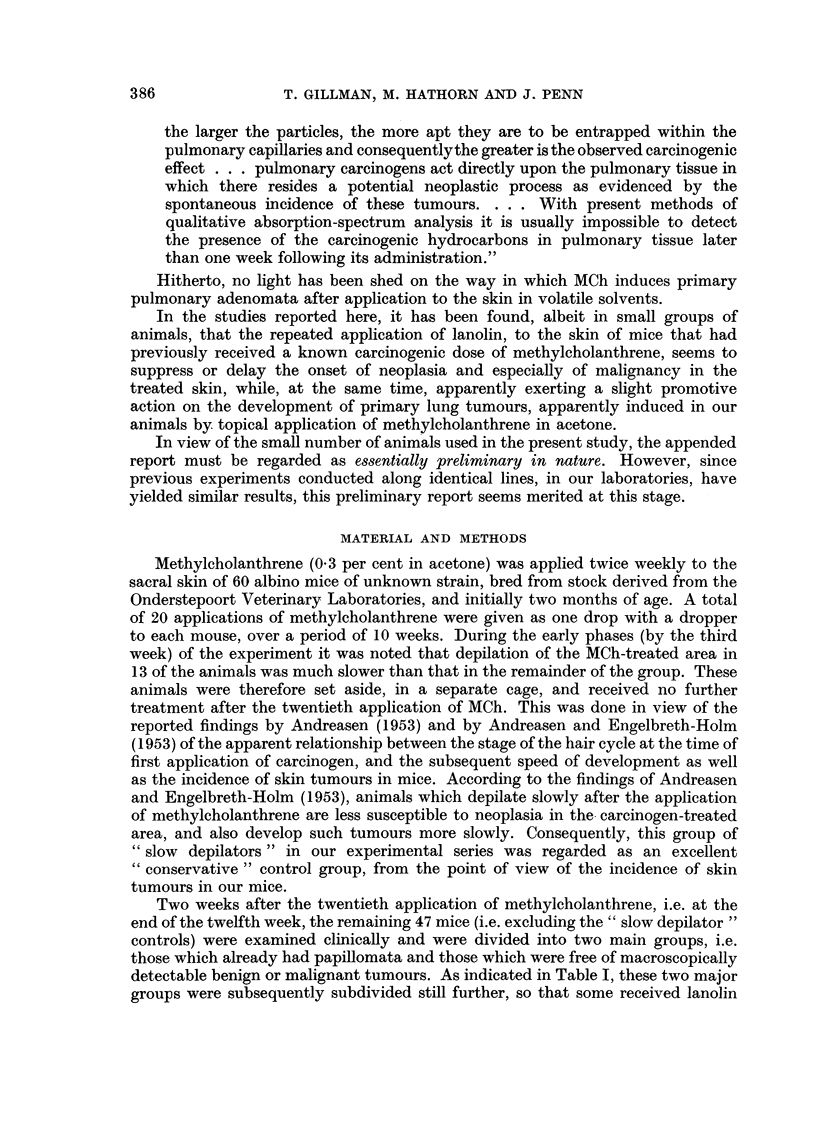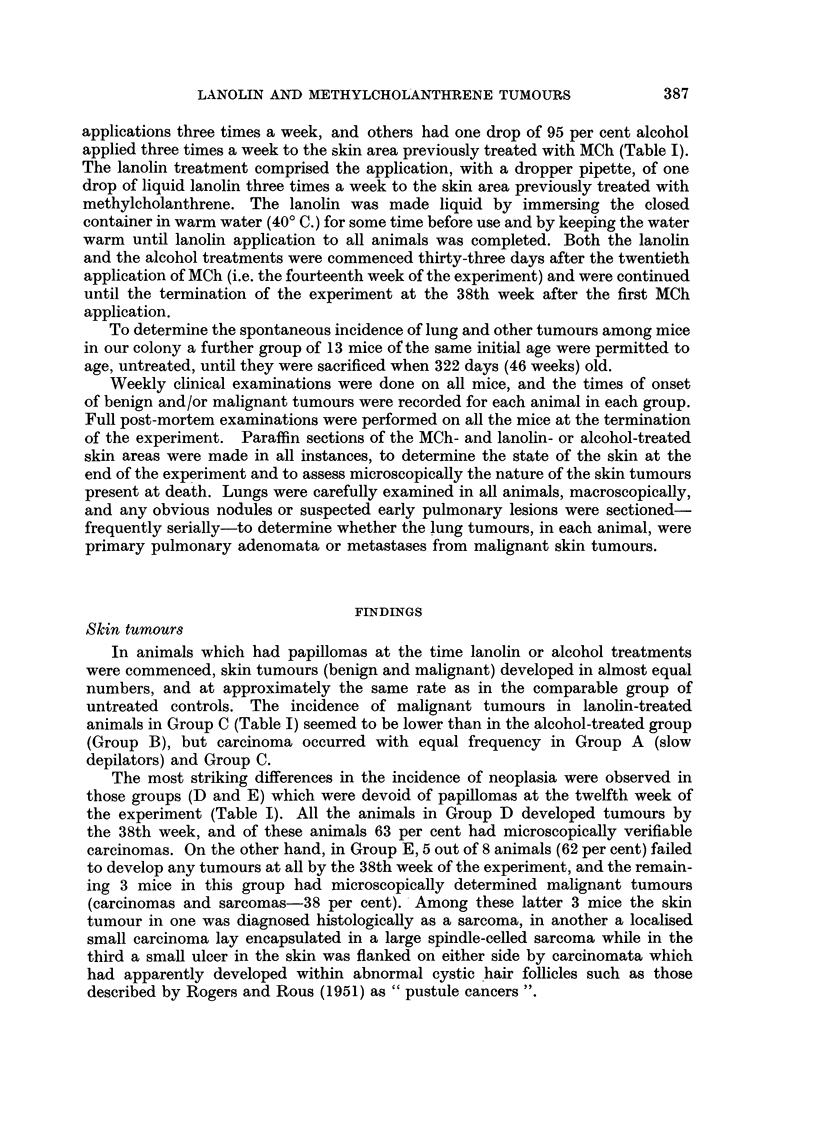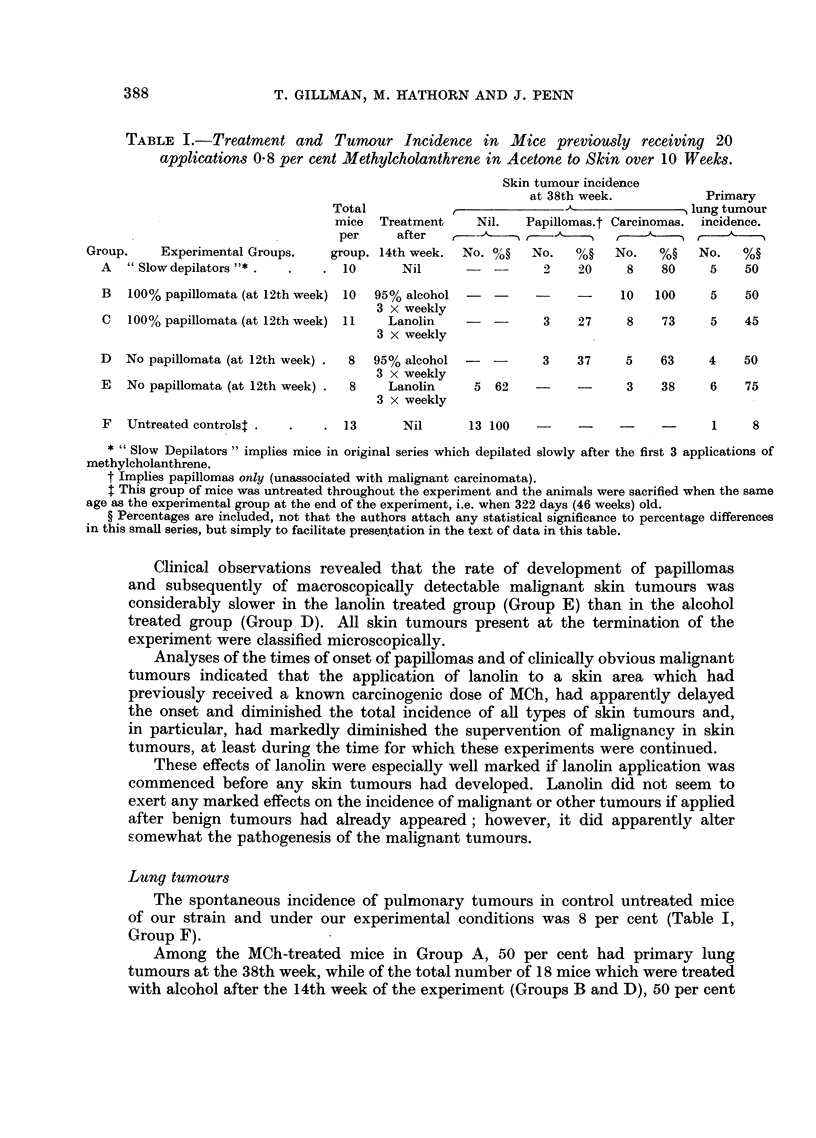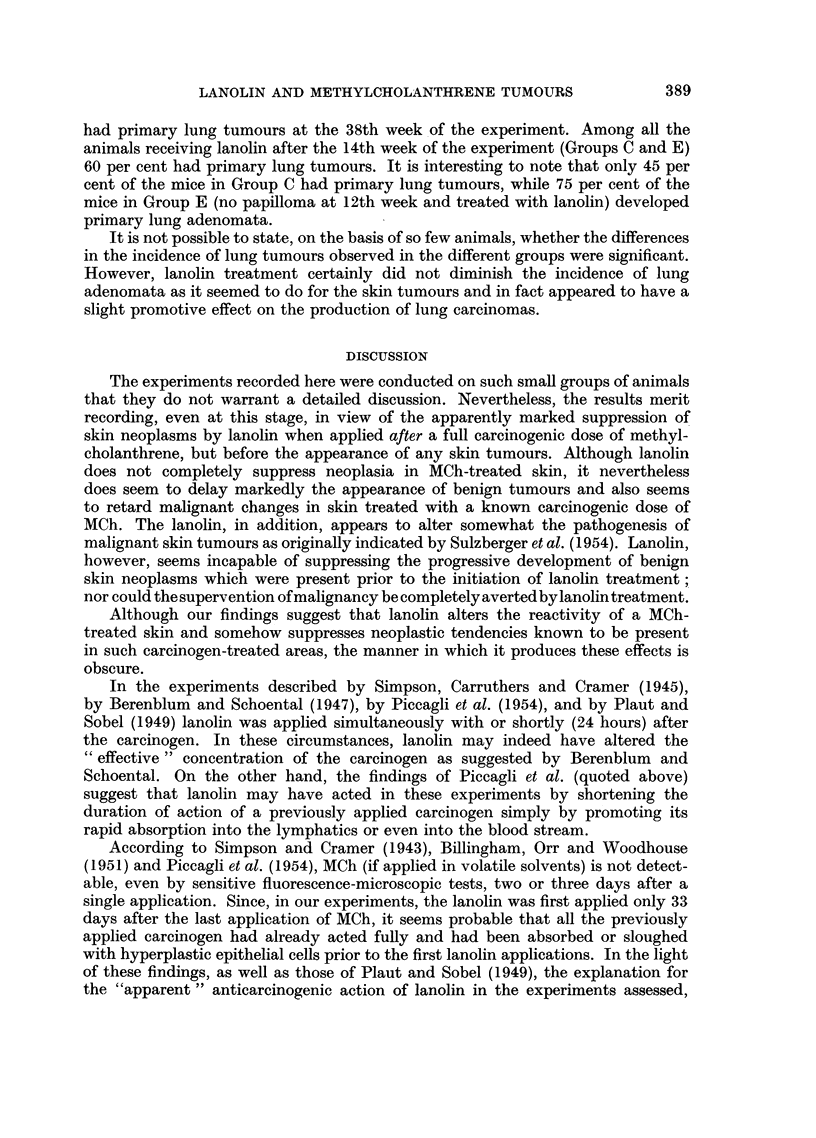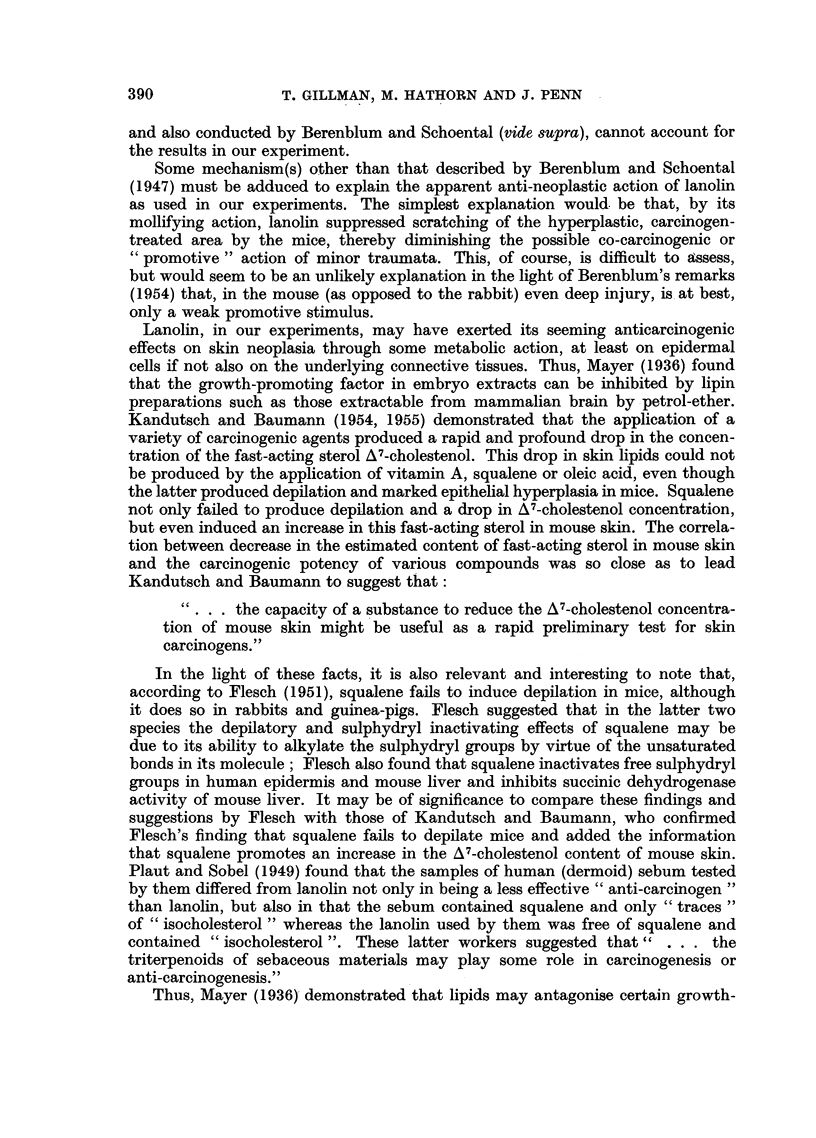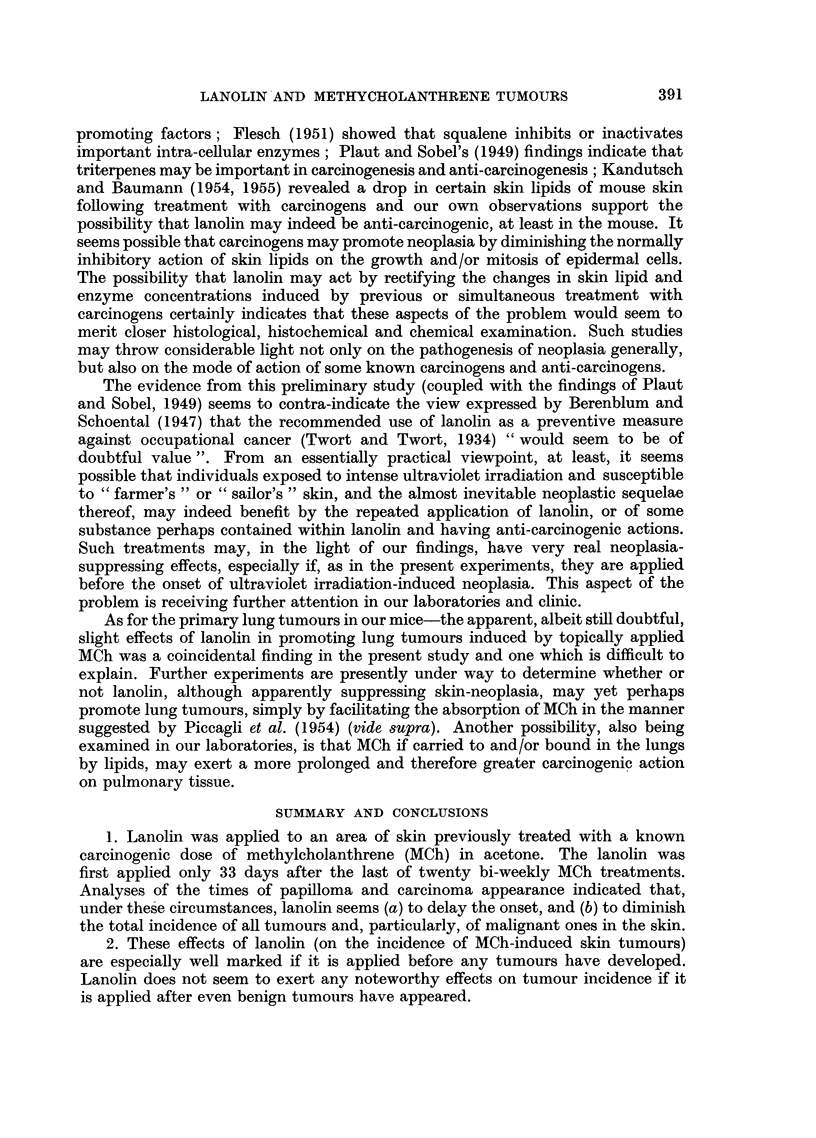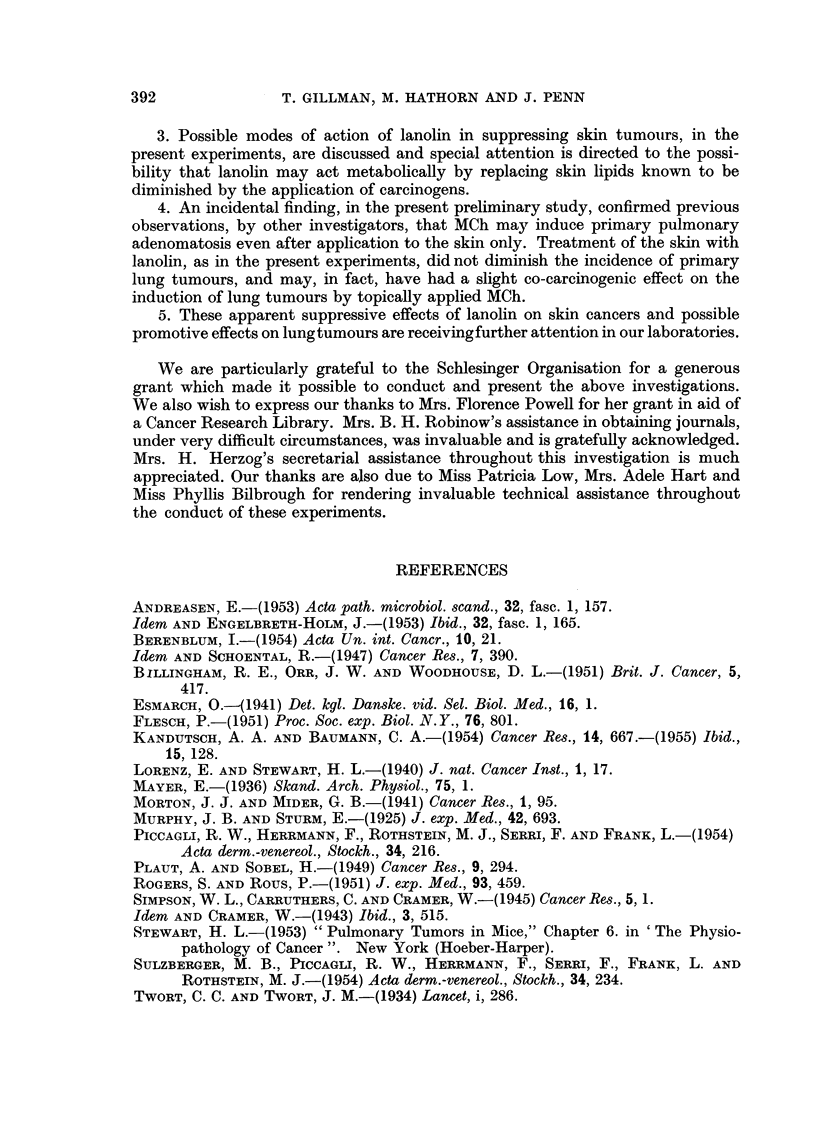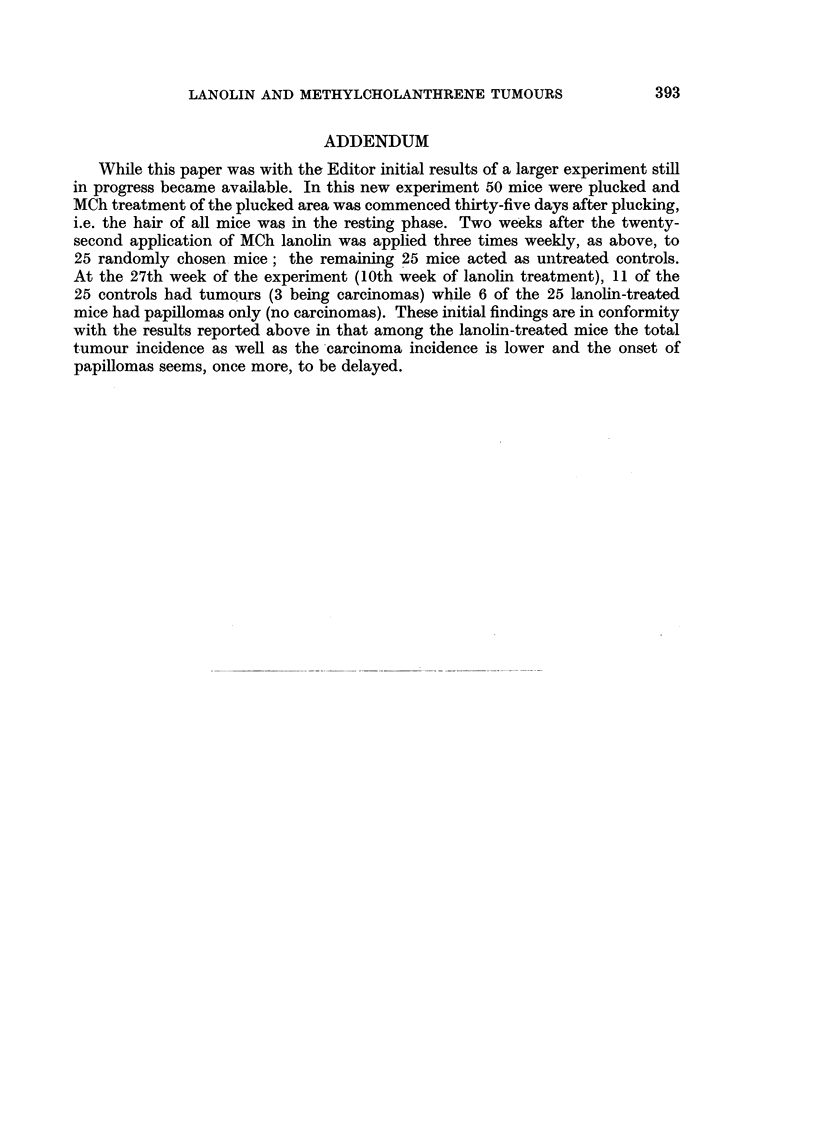# Apparent Paradoxical Effects of Lanolin on Induction of Skin and Lung Tumours by Topically Applied Methylcholanthrene

**DOI:** 10.1038/bjc.1956.44

**Published:** 1956-06

**Authors:** T. Gillman, M. Hathorn, J. Penn


### APPARENT PARADOXICAL EFFECTS OF LANOLIN ON INDUCTION OF SKIN AND LUNG TUMOURS BY TOPICALLY APPLIED METHYLCHOLANTHRENE

### A PRELIMINARY REPORT

T. GILLMAN, M. HATHORN AND J. PENN

*From the Department of Physiology and Schlesinger Organisation Medical Research Unit, Faculty of Medicine, University of Natal, Durban, South Africa*

Received for publication February 7, 1956.

INTEREST in the effects of lanolin on the induction of tumours of the skin by locally applied carcinogens has waned somewhat since the publication of the study by Berenblum and Schoental (1947). These authors reviewed the literature indicating that lanolin has an inhibitory effect on the induction of skin tumours by topically applied methylcholanthrene. They reported, moreover, that the inability to induce skin tumours by the topical application of methylcholanthrene dissolved in lanolin is entirely attributable to the fact that, in such a non-volatile solvent, the effective concentration of the carcinogen is much lower than when dissolved in volatile solvents, such as benzene or acetone. To prove their point of view, Berenblum and Schoental showed that by increasing the concentration of methylcholanthrene (MCh) in lanolin the total tumour yield was " at least as great as in the control group " (treated with MCh or 9,10-dimethyl-1,2-benzanthracene in benzene). They concluded that :—

> " . . . provided the concentration of the carcinogen is high enough, lanolin serves as an exceptionally favourable medium for *facilitating* carcinogenesis. In view of this, its recommended use as a preventive measure against occupational cancer would seem to be of doubtful value. Finally, conclusions regarding the mechanism of carcinogenesis, based on the belief that the inhibitory effect of lanolin is specific, must be deemed invalid in the absence of more substantial supports." (Our italics.)

A justifiable criticism of these results, reported by Berenblum and Schoental, is that although they administered MCh in 3 per cent solution in lanolin, in the one experiment, they failed to conduct a control experiment in which 3 per cent of MCh in benzene was used as well.

Plaut and Sobel (1949) compared the carcinogenicity of MCh when dissolved in benzene, lard, human sebum (from dermoid cysts) and lanolin. Methylcholanthrene dissolved in lanolin failed to induce skin neoplasms, while MCh applied in the equally non-volatile vehicles, lard or sebum, did, albeit more slowly than when benzene was the solvent. Plaut and Sobel consequently concluded that the absence of neoplasms, in mice painted with MCh in lanolin, could not be attributed to the non-volatile character of the lanolin, as claimed by Berenblum and Schoental (1947). Plaut and Sobel having thus failed to confirm Berenblum and Schoental's findings that lanolin had no particular anticarcinogenic actions, suggested that their results supported earlier claims for some special "anti-carcinogenic" action for lanolin, at least in the mouse.

Piccagli *et al.* (1954) reported that :

> " Interspersed applications of lanolin diminish the total exposure of the skin to methylcholanthrene, while interspersed applications of glycerin or petrolatum increase the persistence of the available carcinogen."

Subsequently, Sulzberger *et al.* (1954) also reported that :

> " A high percentage of mice painted with methylcholanthrene in *lanolin* developed tumours which appeared late, but most of which immediately presented the characteristics of malignancy. This late development of ' carcinoma d'emblée ' differs distinctly from the character of tumour formation observed after the application of methylcholanthrene solutions in benzene."

Thus, as far as the action of lanolin in relation to the induction of skin carcinoma is concerned, there now exists considerable doubt as to, firstly, the possible suppressive effect of lanolin on tumour induction and, secondly, the mode of action of this substance on methylcholanthrene absorption and the duration of MCh action on the skin when applied in lanolin.

Lung tumours (primary pulmonary adenomatosis) have also been induced by the topical application of tar (Murphy and Sturm, 1925), or the oral administration and/or intravenous injection of methylcholanthrene (Lorenz and Stewart, 1940 ; Esmarch, 1941 ; Morton and Mider, 1941). Murphy and Sturm (1925), in discussing the possible mode of production of lung tumours by coal tar, when applied repeatedly to different skin fields in rotation, and in the absence of associated skin tumours, considered several possibilities. Firstly, that the lung tumours were metastases—this was excluded by their experimental procedure, in which skin tumours were avoided ; secondly, they entertained the possibility that the lung tumours in their experimental animals treated with tar may have been spontaneous —this was excluded by the fact that although their strain of mice had a high spontaneous incidence of primary lung cancers, the age of their experimental animals was lower than that at which a high incidence of spontaneous primary lung tumours could be ordinarily anticipated ; the third possibility considered by Murphy and Sturm (1925) was " that the particles (of carcinogen) get into the lungs through lymphatics " (following the absorption of the carcinogen from the skin, or from the alimentary tract, if it had been licked off the skin). These authors regarded this possibility as " a little far-fetched ", since, they argued, " in either case the tar would have to pass one set of lymph glands in case it was to reach the lungs ". Murphy and Sturm (1925) concluded that, since repeated application of tar greatly reduces the resistance of animals to transplanted cancers, that treatment with this carcinogen promoted carcinogenic action of inhaled ordinarily non-carcinogenic but irritating foreign particles of sawdust or wood shavings.

Stewart (1953) reviewed much of the literature relating to the experimental production of primary lung tumours in mice. He states that :

> " The method of intravenous injection is one of the best ways to test exogenous pulmonary carcinogens. Here, the particle size is important, for the larger the particles, the more apt they are to be entrapped within the pulmonary capillaries and consequently the greater is the observed carcinogenic effect . . . pulmonary carcinogens act directly upon the pulmonary tissue in which there resides a potential neoplastic process as evidenced by the spontaneous incidence of these tumours. . . . With present methods of qualitative absorption-spectrum analysis it is usually impossible to detect the presence of the carcinogenic hydrocarbons in pulmonary tissue later than one week following its administration."

Hitherto, no light has been shed on the way in which MCh induces primary pulmonary adenomata after application to the skin in volatile solvents.

In the studies reported here, it has been found, albeit in small groups of animals, that the repeated application of lanolin, to the skin of mice that had previously received a known carcinogenic dose of methylcholanthrene, seems to suppress or delay the onset of neoplasia and especially of malignancy in the treated skin, while, at the same time, apparently exerting a slight promotive action on the development of primary lung tumours, apparently induced in our animals by topical application of methylcholanthrene in acetone.

In view of the small number of animals used in the present study, the appended report must be regarded as *essentially preliminary in nature.* However, since previous experiments conducted along identical lines, in our laboratories, have yielded similar results, this preliminary report seems merited at this stage.

### MATERIAL AND METHODS

Methylcholanthrene (0·3 per cent in acetone) was applied twice weekly to the sacral skin of 60 albino mice of unknown strain, bred from stock derived from the Onderstepoort Veterinary Laboratories, and initially two months of age. A total of 20 applications of methylcholanthrene were given as one drop with a dropper to each mouse, over a period of 10 weeks. During the early phases (by the third week) of the experiment it was noted that depilation of the MCh-treated area in 13 of the animals was much slower than that in the remainder of the group. These animals were therefore set aside, in a separate cage, and received no further treatment after the twentieth application of MCh. This was done in view of the reported findings by Andreasen (1953) and by Andreasen and Engelbreth-Holm (1953) of the apparent relationship between the stage of the hair cycle at the time of first application of carcinogen, and the subsequent speed of development as well as the incidence of skin tumours in mice. According to the findings of Andreasen and Engelbreth-Holm (1953), animals which depilate slowly after the application of methylcholanthrene are less susceptible to neoplasia in the carcinogen-treated area, and also develop such tumours more slowly. Consequently, this group of " slow depilators " in our experimental series was regarded as an excellent " conservative " control group, from the point of view of the incidence of skin tumours in our mice.

Two weeks after the twentieth application of methylcholanthrene, i.e. at the end of the twelfth week, the remaining 47 mice (i.e. excluding the " slow depilator " controls) were examined clinically and were divided into two main groups, i.e. those which already had papillomata and those which were free of macroscopically detectable benign or malignant tumours. As indicated in Table I, these two major groups were subsequently subdivided still further, so that some received lanolin applications three times a week, and others had one drop of 95 per cent alcohol applied three times a week to the skin area previously treated with MCh (Table I). The lanolin treatment comprised the application, with a dropper pipette, of one drop of liquid lanolin three times a week to the skin area previously treated with methylcholanthrene. The lanolin was made liquid by immersing the closed container in warm water (40° C.) for some time before use and by keeping the water warm until lanolin application to all animals was completed. Both the lanolin and the alcohol treatments were commenced thirty-three days after the twentieth application of MCh (i.e. the fourteenth week of the experiment) and were continued until the termination of the experiment at the 38th week after the first MCh application.

To determine the spontaneous incidence of lung and other tumours among mice in our colony a further group of 13 mice of the same initial age were permitted to age, untreated, until they were sacrificed when 322 days (46 weeks) old.

Weekly clinical examinations were done on all mice, and the times of onset of benign and/or malignant tumours were recorded for each animal in each group. Full post-mortem examinations were performed on all the mice at the termination of the experiment. Paraffin sections of the MCh- and lanolin- or alcohol-treated skin areas were made in all instances, to determine the state of the skin at the end of the experiment and to assess microscopically the nature of the skin tumours present at death. Lungs were carefully examined in all animals, macroscopically, and any obvious nodules or suspected early pulmonary lesions were sectioned—frequently serially—to determine whether the lung tumours, in each animal, were primary pulmonary adenomata or metastases from malignant skin tumours.

### FINDINGS

### *Skin tumours*

In animals which had papillomas at the time lanolin or alcohol treatments were commenced, skin tumours (benign and malignant) developed in almost equal numbers, and at approximately the same rate as in the comparable group of untreated controls. The incidence of malignant tumours in lanolin-treated animals in Group C (Table I) seemed to be lower than in the alcohol-treated group (Group B), but carcinoma occurred with equal frequency in Group A (slow depilators) and Group C.

The most striking differences in the incidence of neoplasia were observed in those groups (D and E) which were devoid of papillomas at the twelfth week of the experiment (Table I). All the animals in Group D developed tumours by the 38th week, and of these animals 63 per cent had microscopically verifiable carcinomas. On the other hand, in Group E, 5 out of 8 animals (62 per cent) failed to develop any tumours at all by the 38th week of the experiment, and the remaining 3 mice in this group had microscopically determined malignant tumours (carcinomas and sarcomas—38 per cent). Among these latter 3 mice the skin tumour in one was diagnosed histologically as a sarcoma, in another a localised small carcinoma lay encapsulated in a large spindle-celled sarcoma while in the third a small ulcer in the skin was flanked on either side by carcinomata which had apparently developed within abnormal cystic hair follicles such as those described by Rogers and Rous (1951) as " pustule cancers ".

TABLE I.—*Treatment and Tumour Incidence in Mice previously receiving* 20 *applications* 0·8 *per cent Methylcholanthrene in Acetone to Skin over* 10 *Weeks.*

| Group. | Experimental Groups. | Total mice per group. | Treatment after 14th week. | Skin tumour incidence at 38th week. Nil. No. | %§ | Papillomas.† No. | %§ | Carcinomas. No. | %§ | Primary lung tumour incidence. No. | %§ |
|---|---|---|---|---|---|---|---|---|---|---|---|
| A | " Slow depilators "* . . . | 10 | Nil | — | — | 2 | 20 | 8 | 80 | 5 | 50 |
| B | 100% papillomata (at 12th week) | 10 | 95% alcohol 3 × weekly | — | — | — | — | 10 | 100 | 5 | 50 |
| C | 100% papillomata (at 12th week) | 11 | Lanolin 3 × weekly | — | — | 3 | 27 | 8 | 73 | 5 | 45 |
| D | No papillomata (at 12th week) . | 8 | 95% alcohol 3 × weekly | — | — | 3 | 37 | 5 | 63 | 4 | 50 |
| E | No papillomata (at 12th week) . | 8 | Lanolin 3 × weekly | 5 | 62 | — | — | 3 | 38 | 6 | 75 |
| F | Untreated controls‡ . . . | 13 | Nil | 13 | 100 | — | — | — | — | 1 | 8 |

* " Slow Depilators " implies mice in original series which depilated slowly after the first 3 applications of methylcholanthrene.

† Implies papillomas *only* (unassociated with malignant carcinomata).

‡ This group of mice was untreated throughout the experiment and the animals were sacrified when the same age as the experimental group at the end of the experiment, i.e. when 322 days (46 weeks) old.

§ Percentages are included, not that the authors attach any statistical significance to percentage differences in this small series, but simply to facilitate presentation in the text of data in this table.

Clinical observations revealed that the rate of development of papillomas and subsequently of macroscopically detectable malignant skin tumours was considerably slower in the lanolin treated group (Group E) than in the alcohol treated group (Group D). All skin tumours present at the termination of the experiment were classified microscopically.

Analyses of the times of onset of papillomas and of clinically obvious malignant tumours indicated that the application of lanolin to a skin area which had previously received a known carcinogenic dose of MCh, had apparently delayed the onset and diminished the total incidence of all types of skin tumours and, in particular, had markedly diminished the supervention of malignancy in skin tumours, at least during the time for which these experiments were continued.

These effects of lanolin were especially well marked if lanolin application was commenced before any skin tumours had developed. Lanolin did not seem to exert any marked effects on the incidence of malignant or other tumours if applied after benign tumours had already appeared; however, it did apparently alter somewhat the pathogenesis of the malignant tumours.

*Lung tumours*

The spontaneous incidence of pulmonary tumours in control untreated mice of our strain and under our experimental conditions was 8 per cent (Table I, Group F).

Among the MCh-treated mice in Group A, 50 per cent had primary lung tumours at the 38th week, while of the total number of 18 mice which were treated with alcohol after the 14th week of the experiment (Groups B and D), 50 per cent had primary lung tumours at the 38th week of the experiment. Among all the animals receiving lanolin after the 14th week of the experiment (Groups C and E) 60 per cent had primary lung tumours. It is interesting to note that only 45 per cent of the mice in Group C had primary lung tumours, while 75 per cent of the mice in Group E (no papilloma at 12th week and treated with lanolin) developed primary lung adenomata.

It is not possible to state, on the basis of so few animals, whether the differences in the incidence of lung tumours observed in the different groups were significant. However, lanolin treatment certainly did not diminish the incidence of lung adenomata as it seemed to do for the skin tumours and in fact appeared to have a slight promotive effect on the production of lung carcinomas.

### DISCUSSION

The experiments recorded here were conducted on such small groups of animals that they do not warrant a detailed discussion. Nevertheless, the results merit recording, even at this stage, in view of the apparently marked suppression of skin neoplasms by lanolin when applied *after* a full carcinogenic dose of methylcholanthrene, but before the appearance of any skin tumours. Although lanolin does not completely suppress neoplasia in MCh-treated skin, it nevertheless does seem to delay markedly the appearance of benign tumours and also seems to retard malignant changes in skin treated with a known carcinogenic dose of MCh. The lanolin, in addition, appears to alter somewhat the pathogenesis of malignant skin tumours as originally indicated by Sulzberger *et al.* (1954). Lanolin, however, seems incapable of suppressing the progressive development of benign skin neoplasms which were present prior to the initiation of lanolin treatment; nor could the supervention of malignancy be completely averted by lanolin treatment.

Although our findings suggest that lanolin alters the reactivity of a MCh-treated skin and somehow suppresses neoplastic tendencies known to be present in such carcinogen-treated areas, the manner in which it produces these effects is obscure.

In the experiments described by Simpson, Carruthers and Cramer (1945), by Berenblum and Schoental (1947), by Piccagli *et al.* (1954), and by Plaut and Sobel (1949) lanolin was applied simultaneously with or shortly (24 hours) after the carcinogen. In these circumstances, lanolin may indeed have altered the "effective" concentration of the carcinogen as suggested by Berenblum and Schoental. On the other hand, the findings of Piccagli *et al.* (quoted above) suggest that lanolin may have acted in these experiments by shortening the duration of action of a previously applied carcinogen simply by promoting its rapid absorption into the lymphatics or even into the blood stream.

According to Simpson and Cramer (1943), Billingham, Orr and Woodhouse (1951) and Piccagli *et al.* (1954), MCh (if applied in volatile solvents) is not detectable, even by sensitive fluorescence-microscopic tests, two or three days after a single application. Since, in our experiments, the lanolin was first applied only 33 days after the last application of MCh, it seems probable that all the previously applied carcinogen had already acted fully and had been absorbed or sloughed with hyperplastic epithelial cells prior to the first lanolin applications. In the light of these findings, as well as those of Plaut and Sobel (1949), the explanation for the "apparent" anticarcinogenic action of lanolin in the experiments assessed, and also conducted by Berenblum and Schoental (*vide supra*), cannot account for the results in our experiment.

Some mechanism(s) other than that described by Berenblum and Schoental (1947) must be adduced to explain the apparent anti-neoplastic action of lanolin as used in our experiments. The simplest explanation would be that, by its mollifying action, lanolin suppressed scratching of the hyperplastic, carcinogen-treated area by the mice, thereby diminishing the possible co-carcinogenic or " promotive " action of minor traumata. This, of course, is difficult to assess, but would seem to be an unlikely explanation in the light of Berenblum's remarks (1954) that, in the mouse (as opposed to the rabbit) even deep injury, is at best, only a weak promotive stimulus.

Lanolin, in our experiments, may have exerted its seeming anticarcinogenic effects on skin neoplasia through some metabolic action, at least on epidermal cells if not also on the underlying connective tissues. Thus, Mayer (1936) found that the growth-promoting factor in embryo extracts can be inhibited by lipin preparations such as those extractable from mammalian brain by petrol-ether. Kandutsch and Baumann (1954, 1955) demonstrated that the application of a variety of carcinogenic agents produced a rapid and profound drop in the concentration of the fast-acting sterol $\Delta^7$-cholestenol. This drop in skin lipids could not be produced by the application of vitamin A, squalene or oleic acid, even though the latter produced depilation and marked epithelial hyperplasia in mice. Squalene not only failed to produce depilation and a drop in $\Delta^7$-cholestenol concentration, but even induced an increase in this fast-acting sterol in mouse skin. The correlation between decrease in the estimated content of fast-acting sterol in mouse skin and the carcinogenic potency of various compounds was so close as to lead Kandutsch and Baumann to suggest that :

> " . . . the capacity of a substance to reduce the $\Delta^7$-cholestenol concentration of mouse skin might be useful as a rapid preliminary test for skin carcinogens."

In the light of these facts, it is also relevant and interesting to note that, according to Flesch (1951), squalene fails to induce depilation in mice, although it does so in rabbits and guinea-pigs. Flesch suggested that in the latter two species the depilatory and sulphydryl inactivating effects of squalene may be due to its ability to alkylate the sulphydryl groups by virtue of the unsaturated bonds in its molecule ; Flesch also found that squalene inactivates free sulphydryl groups in human epidermis and mouse liver and inhibits succinic dehydrogenase activity of mouse liver. It may be of significance to compare these findings and suggestions by Flesch with those of Kandutsch and Baumann, who confirmed Flesch's finding that squalene fails to depilate mice and added the information that squalene promotes an increase in the $\Delta^7$-cholestenol content of mouse skin. Plaut and Sobel (1949) found that the samples of human (dermoid) sebum tested by them differed from lanolin not only in being a less effective " anti-carcinogen " than lanolin, but also in that the sebum contained squalene and only " traces " of " isocholesterol " whereas the lanolin used by them was free of squalene and contained " isocholesterol ". These latter workers suggested that " . . . the triterpenoids of sebaceous materials may play some role in carcinogenesis or anti-carcinogenesis."

Thus, Mayer (1936) demonstrated that lipids may antagonise certain growthpromoting factors; Flesch (1951) showed that squalene inhibits or inactivates important intra-cellular enzymes; Plaut and Sobel's (1949) findings indicate that triterpenes may be important in carcinogenesis and anti-carcinogenesis; Kandutsch and Baumann (1954, 1955) revealed a drop in certain skin lipids of mouse skin following treatment with carcinogens and our own observations support the possibility that lanolin may indeed be anti-carcinogenic, at least in the mouse. It seems possible that carcinogens may promote neoplasia by diminishing the normally inhibitory action of skin lipids on the growth and/or mitosis of epidermal cells. The possibility that lanolin may act by rectifying the changes in skin lipid and enzyme concentrations induced by previous or simultaneous treatment with carcinogens certainly indicates that these aspects of the problem would seem to merit closer histological, histochemical and chemical examination. Such studies may throw considerable light not only on the pathogenesis of neoplasia generally, but also on the mode of action of some known carcinogens and anti-carcinogens.

The evidence from this preliminary study (coupled with the findings of Plaut and Sobel, 1949) seems to contra-indicate the view expressed by Berenblum and Schoental (1947) that the recommended use of lanolin as a preventive measure against occupational cancer (Twort and Twort, 1934) " would seem to be of doubtful value ". From an essentially practical viewpoint, at least, it seems possible that individuals exposed to intense ultraviolet irradiation and susceptible to " farmer's " or " sailor's " skin, and the almost inevitable neoplastic sequelae thereof, may indeed benefit by the repeated application of lanolin, or of some substance perhaps contained within lanolin and having anti-carcinogenic actions. Such treatments may, in the light of our findings, have very real neoplasia-suppressing effects, especially if, as in the present experiments, they are applied before the onset of ultraviolet irradiation-induced neoplasia. This aspect of the problem is receiving further attention in our laboratories and clinic.

As for the primary lung tumours in our mice—the apparent, albeit still doubtful, slight effects of lanolin in promoting lung tumours induced by topically applied MCh was a coincidental finding in the present study and one which is difficult to explain. Further experiments are presently under way to determine whether or not lanolin, although apparently suppressing skin-neoplasia, may yet perhaps promote lung tumours, simply by facilitating the absorption of MCh in the manner suggested by Piccagli *et al.* (1954) (*vide supra*). Another possibility, also being examined in our laboratories, is that MCh if carried to and/or bound in the lungs by lipids, may exert a more prolonged and therefore greater carcinogenic action on pulmonary tissue.

### SUMMARY AND CONCLUSIONS

1. Lanolin was applied to an area of skin previously treated with a known carcinogenic dose of methylcholanthrene (MCh) in acetone. The lanolin was first applied only 33 days after the last of twenty bi-weekly MCh treatments. Analyses of the times of papilloma and carcinoma appearance indicated that, under these circumstances, lanolin seems (*a*) to delay the onset, and (*b*) to diminish the total incidence of all tumours and, particularly, of malignant ones in the skin.

2. These effects of lanolin (on the incidence of MCh-induced skin tumours) are especially well marked if it is applied before any tumours have developed. Lanolin does not seem to exert any noteworthy effects on tumour incidence if it is applied after even benign tumours have appeared.

3. Possible modes of action of lanolin in suppressing skin tumours, in the present experiments, are discussed and special attention is directed to the possibility that lanolin may act metabolically by replacing skin lipids known to be diminished by the application of carcinogens.

4. An incidental finding, in the present preliminary study, confirmed previous observations, by other investigators, that MCh may induce primary pulmonary adenomatosis even after application to the skin only. Treatment of the skin with lanolin, as in the present experiments, did not diminish the incidence of primary lung tumours, and may, in fact, have had a slight co-carcinogenic effect on the induction of lung tumours by topically applied MCh.

5. These apparent suppressive effects of lanolin on skin cancers and possible promotive effects on lung tumours are receiving further attention in our laboratories.

We are particularly grateful to the Schlesinger Organisation for a generous grant which made it possible to conduct and present the above investigations. We also wish to express our thanks to Mrs. Florence Powell for her grant in aid of a Cancer Research Library. Mrs. B. H. Robinow's assistance in obtaining journals, under very difficult circumstances, was invaluable and is gratefully acknowledged. Mrs. H. Herzog's secretarial assistance throughout this investigation is much appreciated. Our thanks are also due to Miss Patricia Low, Mrs. Adele Hart and Miss Phyllis Bilbrough for rendering invaluable technical assistance throughout the conduct of these experiments.

### REFERENCES

ANDREASEN, E.—(1953) *Acta path. microbiol. scand.*, **32**, fasc. 1, 157.
*Idem* AND ENGELBRETH-HOLM, J.—(1953) *Ibid.*, **32**, fasc. 1, 165.
BERENBLUM, I.—(1954) *Acta Un. int. Cancr.*, **10**, 21.
*Idem* AND SCHOENTAL, R.—(1947) *Cancer Res.*, **7**, 390.
BILLINGHAM, R. E., ORR, J. W. AND WOODHOUSE, D. L.—(1951) *Brit. J. Cancer*, **5**, 417.
ESMARCH, O.—(1941) *Det. kgl. Danske. vid. Sel. Biol. Med.*, **16**, 1.
FLESCH, P.—(1951) *Proc. Soc. exp. Biol. N.Y.*, **76**, 801.
KANDUTSCH, A. A. AND BAUMANN, C. A.—(1954) *Cancer Res.*, **14**, 667.—(1955) *Ibid.*, **15**, 128.
LORENZ, E. AND STEWART, H. L.—(1940) *J. nat. Cancer Inst.*, **1**, 17.
MAYER, E.—(1936) *Skand. Arch. Physiol.*, **75**, 1.
MORTON, J. J. AND MIDER, G. B.—(1941) *Cancer Res.*, **1**, 95.
MURPHY, J. B. AND STURM, E.—(1925) *J. exp. Med.*, **42**, 693.
PICCAGLI, R. W., HERRMANN, F., ROTHSTEIN, M. J., SERRI, F. AND FRANK, L.—(1954) *Acta derm.-venereol., Stockh.*, **34**, 216.
PLAUT, A. AND SOBEL, H.—(1949) *Cancer Res.*, **9**, 294.
ROGERS, S. AND ROUS, P.—(1951) *J. exp. Med.*, **93**, 459.
SIMPSON, W. L., CARRUTHERS, C. AND CRAMER, W.—(1945) *Cancer Res.*, **5**, 1.
*Idem* AND CRAMER, W.—(1943) *Ibid.*, **3**, 515.
STEWART, H. L.—(1953) " Pulmonary Tumors in Mice," Chapter 6. in ' The Physiopathology of Cancer ". New York (Hoeber-Harper).
SULZBERGER, M. B., PICCAGLI, R. W., HERRMANN, F., SERRI, F., FRANK, L. AND ROTHSTEIN, M. J.—(1954) *Acta derm.-venereol., Stockh.*, **34**, 234.
TWORT, C. C. AND TWORT, J. M.—(1934) *Lancet*, i, 286.

### ADDENDUM

While this paper was with the Editor initial results of a larger experiment still in progress became available. In this new experiment 50 mice were plucked and MCh treatment of the plucked area was commenced thirty-five days after plucking, i.e. the hair of all mice was in the resting phase. Two weeks after the twenty-second application of MCh lanolin was applied three times weekly, as above, to 25 randomly chosen mice ; the remaining 25 mice acted as untreated controls. At the 27th week of the experiment (10th week of lanolin treatment), 11 of the 25 controls had tumours (3 being carcinomas) while 6 of the 25 lanolin-treated mice had papillomas only (no carcinomas). These initial findings are in conformity with the results reported above in that among the lanolin-treated mice the total tumour incidence as well as the carcinoma incidence is lower and the onset of papillomas seems, once more, to be delayed.